# Presence of unsafe chemical impurities, accelerated evaporation of alcohol, and lack of key labeling requirements are risks and concerns for some alcohol-based hand sanitizers and dispenser practices during the COVID-19 pandemic

**DOI:** 10.1371/journal.pone.0265519

**Published:** 2022-03-18

**Authors:** Clyde S. Manuel, Dawn J. Yeomans, Jessica A. Williams, Christopher Fricker, Kaury Kucera, David Light, James W. Arbogast

**Affiliations:** 1 GOJO Industries, Inc., Akron, Ohio, United States of America; 2 Valisure, LLC, New Haven, Connecticut, United States of America; Tianjin University of Traditional Chinese Medicine, CHINA

## Abstract

Alcohol-based hand sanitizers (ABHS) have been an important hand hygiene tool during the COVID-19 pandemic. Recently, ABHS from non-traditional drug manufacturers have entered the market, triggered by a lack of ABHS availability. Some of these ABHS contain high levels of chemical impurities that may be harmful with frequent exposure. Additionally, the use of refillable dispensers designed to accept ABHS from bulk containers allows for mixing and evaporation that may compromise ABHS integrity. To understand the risks associated with low quality ABHS and bulk refilling practices, we collected 77 ABHS samples sourced from community settings (restaurants, grocery stores, etc.) and 40 samples from a single school district. All samples were obtained from bulk refillable dispensers that were in use. Samples were analyzed for alcohol content, chemical impurities, aesthetic qualities, and presence of drug labeling information. Additionally, we performed laboratory-based experiments to determine the impact of dispenser design on alcohol evaporation rates. Over 70% of samples for which photos were available showed lack of essential labeling information, including missing “Drug Facts Labels”. For ABHS samples acquired from community settings, nearly 14% of samples had visible impurities, and over 30% of samples had concentrations of acetal and acetaldehyde in excess of FDA interim limits. Subpotent ethanol concentrations were observed in 9.09% and 82.05% of samples from community settings and the school district, respectively, with the school district sample results being associated with dispenser misuse. Laboratory-based experiments show dispenser design significantly impacts the rate of ethanol evaporation of ABHS products, especially if stored in open refillable dispensers without an internal reservoir. This study demonstrates risks associated with use of inferior ABHS and bulk refilling practices. Regulatory agencies should issue guidance on best practices in community settings to ensure the integrity of ABHS as an essential public health tool to prevent the spread of COVID-19 and other transmissible diseases.

## Introduction

The COVID-19 pandemic has had a significant global impact on public health. As of November 2021, cumulative deaths attributed to COVID-19 were over 5 million globally and over 750,000 in the United States [1]. While the development of multiple effective vaccines against COVID-19 has been a significant milestone in the global fight against this pandemic [2], non-pharmaceutical interventions (NPIs) such as social distancing, mask use, and hand hygiene, continue to be important tools for preventing the spread of COVID-19 [2].

Hand hygiene is widely accepted as an important NPI for prevention of disease transmission of COVID-19. Previous studies have linked proper hand hygiene to a significant reduction in the spread of transmissible disease [3]. According to the Centers for Disease Control and Prevention (CDC), SARS-CoV-2, the causative viral agent of COVID-19, primarily spreads through direct person-to-person contact, indirect contact, or droplet contact [4]. As a method to prevent transmission of SARS-CoV-2, the CDC recommends performing proper hand hygiene by washing hands with soap and water for at least 20 seconds [4]. The US CDC COVID-19 public health protection guidelines include the recommendation that “If soap and water are not readily available, use a hand sanitizer that contains at least 60% alcohol” [4]. Additionally, the World Health Organization (WHO) also recommends that individuals “regularly and thoroughly clean your hands with an alcohol-based hand rub or wash them with soap and water” [5] and that “hand rub dispensers, should be put in prominent places around the workplace and be made accessible to all staff, contractors, clients or customers, and visitors” [6]. These public health messages have also been adopted at the local level. For example, The Ohio Department of Health encourages individuals to “use alcohol-based hand sanitizer when soap and water are unavailable” [7].

Given the importance of ABHS to reduce COVID-19 spread, sales of ABHS globally grew exponentially during the initial weeks of the COVID-19 pandemic, causing a shortage of ABHS supply in many markets. To alleviate these shortages, many countries that regulate ABHS as drugs or therapeutics instituted temporary production policies allowing new suppliers of ABHS easy market entry. Canada, The United States, and Australia, are just three examples of major countries instituting such policies [8–10].

To increase supply of ABHS in the United States, the US Food and Drug Administration (FDA), who regulate ABHS as Over-The-Counter (OTC) Drugs, issued a Guidance Document in March 2020 titled “Temporary Policy for Preparation of Certain Alcohol-Based Hand Sanitizer Products During the Public Health Emergency (COVID-19)—Guidance for Industry” [10]. This document provided guidance for non-drug manufacturers (e.g., distilleries, fuel manufacturers) for the preparation and distribution of ABHS products for the public’s use during the COVID-19 public health emergency and was intended to alleviate challenges associated with sourcing ABHS. On October 12, 2021, FDA announced the withdrawal of this guidance document approximately 19 months after it was initiated, which will require temporary ABHS producers to fully comply with federal OTC drug production regulations, beginning January 1, 2022. FDA cited ABHS supplies returning to pre-pandemic levels as the main reason for withdrawal [11].

For temporary manufacturers of ABHS, the FDA guidance document identified several critical considerations for production of ABHS, including:

1. Alcohol source must be either ethanol not less than 94.9% by volume, or United States Pharmacopeia (USP) grade Isopropyl Alcohol (IPA)
2. Ethanol produced using fermentation and distillation processes typically used for consumable goods may be used if the alcohol meets interim impurity levels set by the FDA
3. Ethanol produced in facilities normally producing fuel or technical grade alcohol may be used provided the alcohol meets interim impurity levels set by the FDA
4. Ingredients exactly matching the WHO formulation created for hospitals in developing, resource limited countries [12], and
5. Finished products are labeled in accordance with criteria established by FDA in their temporary policy (i.e., drug facts label, name, address, and contact information for manufacturer)

As a result of FDA’s temporary policy, ABHS produced by non-traditional drug manufacturers rapidly entered the market to alleviate supply concerns. Unfortunately, many of these ABHS products were produced with materials containing elevated levels of impurities of concern, such as methanol, benzene, acetal, and acetaldehyde. In response to these products, FDA has compiled a list of hand sanitizers consumers should not use [13]. As of November 2021, the list stands at more than 250 products. While many of these products have been voluntarily recalled by the ABHS manufacturer, in some situations, FDA has issued warning letters to the manufacturer to facilitate product withdrawal from the market. In addition to FDA’s findings, there are other reports that demonstrate concerns with many of these ABHS products. For example, an independent laboratory analyzed 260 bottles of ABHS from 168 brands and found that 21 bottles (8%) contained benzene above 2 ppm [14], which is FDA’s temporary threshold for benzene in their temporary ABHS policy [10]. On October 4, 2021, FDA issued a statement adding a specific brand of ABHS, which had the highest levels of impurities in the aforementioned independent laboratory report, to the list of hand sanitizers consumer should not use due to FDA’s confirmation of benzene, and other contaminants beyond acceptable levels [13]. In a similar analysis of 42 commercially available ABHS products in Canada, 11 products were non-compliant with interim Health Canada guidelines, specifically for acetaldehyde concentrations [15]. Collectively, these results highlighted the potential for ABHS produced under these temporary policies to contain unsafe levels of potentially harmful impurities.

At the same time ABHS produced under FDA’s temporary policy entered the market, a dramatic increase in ABHS dispenser installations occurred. Dispensers of all types were installed in community settings (e.g., retail spaces, healthcare, grocery stores, foodservice establishments) to facilitate ABHS use by individuals. Many of these dispensers were of a design allowing the dispenser reservoir or the primary package itself to be easily refilled through use of large -volume containers of ABHS. These dispensers, often called “bulk refillable” dispensers, are not recommended for use in soap dispensing in healthcare settings due to their propensity to become contaminated with pathogenic bacteria when “topped off” [16]. As outlined by CDC, when used for dispensing of ABHS, these bulk dispensers have “potential safety risks…including inadvertent contamination, reduced effectiveness from the evaporation of alcohol, and irritant effects from mixing [of] formulations” [17]. Currently, the potential risks of ABHS use in bulk dispensers are not well studied.

In this study, we collected samples from community settings, including schools, to better characterize potential health risks associated with low quality ABHS and bulk refilling practices of dispensers. We assessed the product quality and safety profile of ABHS encountered in community settings during the COVID-19 pandemic. Additionally, we characterized the alcohol content of ABHS stored in bulk refillable dispensers in the public, and then used laboratory-based experiments to determine the impact of bulk refillable dispenser type and ABHS formula on alcohol evaporation rates. Collectively, the data presented in our study show that ABHS from bulk refillable dispensers in community settings: 1) contain visual impurities (e.g., turbidity or particulates) and lack appropriate required labeling on consumer facing dispensers; 2) contain levels of potentially harmful impurities in excess of interim federal guidance; 3) contain subpotent concentrations of ethanol, which may result in an ineffective product, and 4) have a high rate of alcohol evaporation due to dispenser design and improper use, especially when bulk refillable dispensers are used.

## Materials and methods

### Description of locations sampled

**Fig 1:**
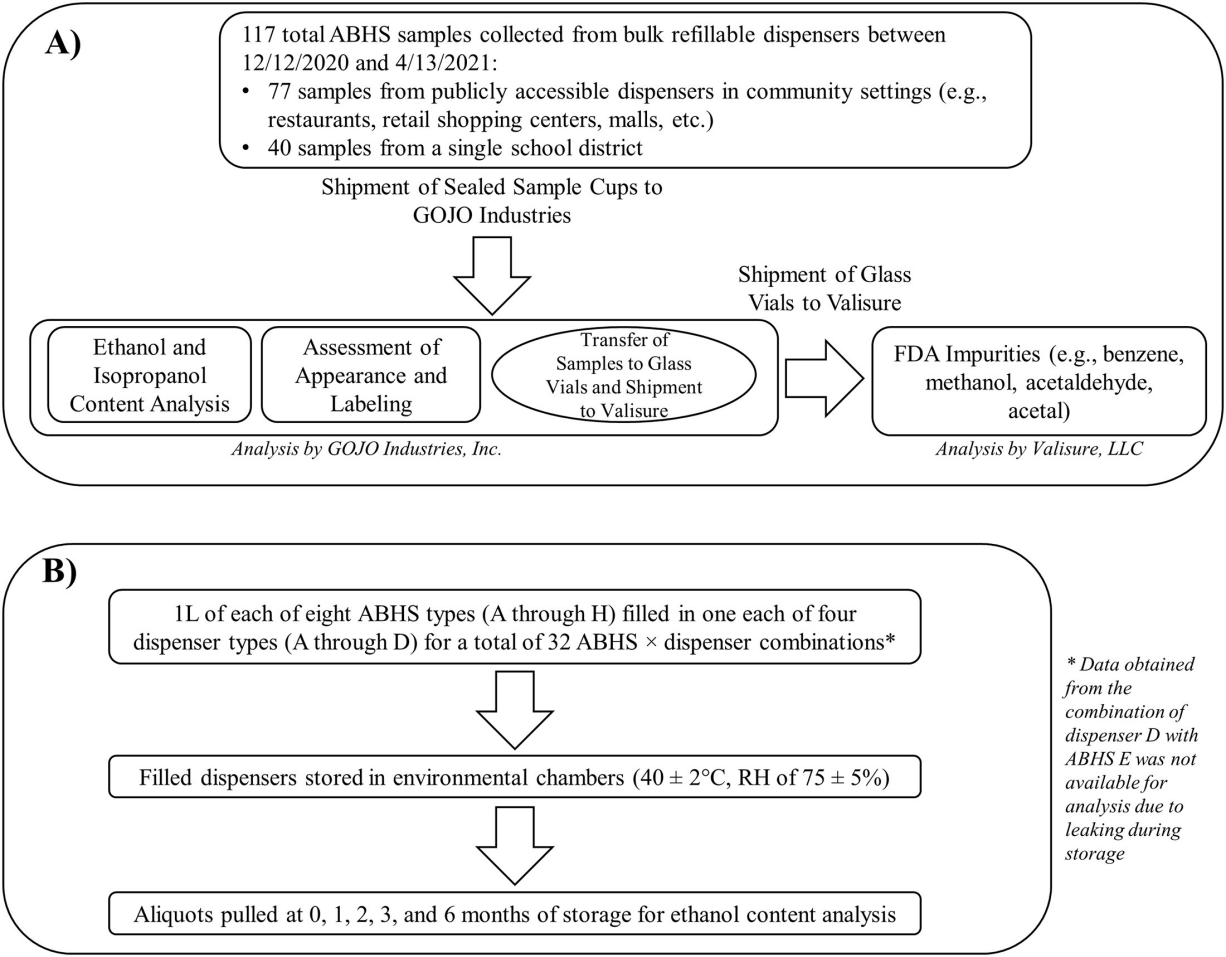
Flow chart of experimental design. Panel A: Field sampling of ABHS from bulk refillable dispensers. Panel B: Evaluation of rate of ethanol evaporation of dispenser and ABHS under accelerated stability conditions.

A total of 117 ABHS samples from bulk refillable dispensers were collected in this study. Seventy-seven samples were randomly acquired from a variety of community settings (e.g., restaurants, retail shopping centers, malls, schools, office buildings, grocery stores, convenience stores, and fitness centers) from several major metropolitan areas across the United States. An additional 40 samples were acquired from a single school district in South Carolina. Samples were collected between December 12, 2020 and April 13, 2021. Sample collectors were instructed to only sample from bulk refillable dispensers that were actively in-use and publicly accessible. Samples out of scope of this research and therefore excluded from analysis include samples obtained from individual bottles and unopened refills, samples formulated without alcohol as an active ingredient, and samples that were not hand sanitizers (e.g., soap). Detailed information on each sample is provided in S1 Table. ABHS samples collected from public settings were not used for laboratory-based ethanol evaporation experiments (see section “Ethanol evaporation under accelerated stability test conditions”). A visual flow chart representing experiments performed in this study is presented in Fig 1.

### Sample collection

Sample collectors were trained to aseptically dispense a minimum of 20 mL of ABHS from bulk dispensers directly into sterile 120 mL medical grade plastic sample cups with screw cap construction (Parter Medical Products, Carson CA). These cups have been previously verified for long term storage of ABHS at room temperature (20°C) and accelerated conditions (40°C) in accordance with drug stability testing guidance issued by the FDA [18]. At the point of sampling, sample collectors recorded the date, time, location, and dispenser type. Additionally, sample collectors were instructed to record any relevant label content located on or within each dispenser, including the product’s “Drug Facts Label”, lot numbers, expiration dates, and other product information. Sample collectors were also asked to collect photos of each dispenser in the field, including the exterior and interior (refill portion) of each dispenser. After collecting samples, the person tightly secured the lids of the sample cup, and wrapped lids with strips of Parafilm (Bemis Company, Inc., Neenah WI) to provide additional protection against evaporation and spilling during transit. All samples were shipped at ambient conditions to GOJO laboratories within one week of collection, except for the single school district samples collected from South Carolina which were shipped within three weeks of the final collection date. Upon receipt, samples intended for FDA impurity analysis were transferred to 20 mL glass screw cap vials (item 03-337-4, Thermo Fisher Scientific, Waltham, MA) prior to shipment at ambient conditions to Valisure, LLC (New Haven, CT) for analysis. Alcohol analysis was performed at GOJO laboratories, while FDA impurity analysis was performed at Valisure laboratories.

### Visual evaluation of samples

ABHS samples were evaluated for color and appearance according to internally developed protocols based on ASTM D1500 (color) [19] and ASTM E2680 (appearance) [20]. In summary, trained technicians evaluated test samples in comparison with a reference standard (in this case, a finished product of PURELL^®^ Advanced Gel, GOJO Industries, Akron, OH). Test samples were evaluated in a well illuminated area free of incidental interference, and were evaluated for color (e.g., colorless, yellow, white) and appearance (e.g., gel, liquid, homogeneous, cloudy, particulates).

### FDA impurity analysis

The method USP <467> Residual Solvents Procedure A was modified from flame ionization detection (FID) to mass spectrometry (MS) detection for benzene in hand sanitizers. The sample preparation and headspace (HS) gas chromatography (GC) methods were also validated for liquid and gel formulations and to allow shorter run time. Identification of analytes is based on the retention time and mass spectral m/z matching to certified reference standard material (Sigma-Aldrich, St. Louis, MO). Analyte quantification is performed by comparing peak area of benzene in a sample to an 8-point calibration curve from 0.02% to up to 200% the interim limit. The lower limit of quantitation (LLOQ) for each analyte is three times the limit of detection plus the measurement uncertainty of 15% for each analyte. These values range from 0.02 to 9.7 ppm depending on the analyte, and specific values are provided in S1 Table.

Agilent G1530A Gas Chromatography (GC) system equipped with 5973 Mass Selective Detector (MS) was utilized for sample analysis, and a DBSelect 624 UI, 60 m × 0.32 mm × 1.8 μm GC column (Agilent Technology, Santa Clara, CA) was used to resolve analytes. Acetonitrile (LC-MS Grade, Honeywell, Muskegon, MI) was used for sample preparation. Samples were prepared gravimetrically to approximately 10% w/w in acetonitrile to a fixed volume and centrifuged prior to analysis.

Standard of benzene (99.8% purity) and isotopic labeled benzene standard (d3-, 99.8% purity) was used for calibration, continuing calibration verification, retention time verification, and recovery checking. Certified reference material USP Class 1 residual solvents mixture was used for calibration confirmation (USP, Rockville, MD). All volumetric glassware used were Class A certified. Ultra-high purity helium carrier gas was certified as 99.999% pure (The AERO ALL-GAS Co., Hartford, CT).

### Alcohol content analysis

Analysis of alcohol content of ABHS samples was performed using one of two methods. Where possible, a quantitative simultaneous analysis of ethanol and isopropanol by capillary gas chromatography was performed as the primary method of analysis. This method is based on a GC-FID method and validated per guidelines in USP <1225>. The ethanol and isopropanol were analyzed using an Agilent 6890N series gas chromatograph (GC) (Agilent Technology, Santa Clara, CA) equipped with a split/splitless inlet and flame ionization detector (FID). The analytical column used was a ZB-Bioethanol Capillary Column with the dimensions of 15 m x 0.25 mm i.d. and a 1.00 μm film thickness (7EG-G020-22, Phenomenex, Torrance, CA). The split/splitless inlet required a 4.00 mm low pressure drop wool inlet liner (5183–4647, Restek, Bellefonte, PA) to improve repeatability of the method and was replaced at every calibration of the instrument. Ultra-high purity hydrogen was used as the carrier gas and fuel for the flame, supplied from a Parker Balston Hydrogen Generator (Parker-Hannifin Corporation, Mayfield Heights, Ohio). High purity nitrogen was used as the makeup gas and high purity compressed air utilized as the oxidizer for the FID. GC vials and caps compatible with the autosampler (7683B, Agilent Technology, Santa Clara, CA) were used for the standard preparations. The method is calibrated with multiple injections of an ethanol/isopropanol standard and use n-propanol as an internal standard. The standard and control samples were created using the certified reference standard ethanol, USP grade absolute 200 proof (111000200, Aaper Alcohol and Chemical Co, Shelbyville, KY), Isopropanol (278475-1L, Sigma-Aldrich, St. Louis, MO) and n-propanol (279544-2L, Sigma-Aldrich, St. Louis, MO). ABHS samples were diluted to 42% ethanol for analysis. A control sample was analyzed after every 8 injections to demonstrate system suitability. The lower limit of quantitation (linear range) of the assay for ethanol was 10.50% (w/w), with a lower limit of detection of 0.77% (w/w). The lower limit of quantitation (linear range) of the assay for isopropanol was 0.30% (w/w).

Due to the sample matrix associated inhibition observed in ABHS samples from schools in the South Carolina school district, an alternative method of ethanol concentration analysis utilizing a DMA 5000 density meter (Anton Paar GmbH, Graz, Austria) was performed. In this method, samples were held at ambient conditions (20°C) during analysis. 1.0 mL of each sample was loaded into the density meter, which then calculates ethanol concentration in percentage by volume according to the International Alcoholometric Tables issued by the International Organisation of Legal Metrology (OIML) according to The International Temperature Scale of 1990. This approach was validated to be accurate to within 0.007% (v/v) ethanol content.

### Ethanol evaporation under accelerated stability test conditions

To determine the impact of dispenser type and ABHS type on ethanol evaporation rate, a series of laboratory experiments based on standard drug stability test procedures with environmental chambers to simulate long term storage were performed. No refillable hand sanitizer dispensers or bulk ABHS obtained from community settings were used for this set of laboratory-based experiments. Rather, a variety of easily obtained unopened refillable hand sanitizer dispensers and bulk alcohol-based hand sanitizers were purchased from e-commerce websites. Dispensers purchased were of varying construction (e.g., refillable exposed basin, refillable internal reservoir, manual vs touch free dispense). ABHS formats purchased included gel, foam, and WHO (liquid) based formats, and multiple brands for each format were purchased. As controls, two sealed 1,200mL dispenser refills of ABHS products from GOJO Inc., (Akron, OH) were used, including PURELL^®^ Healthcare Advanced Ultra Nourishing Hand Sanitizer Foam and PURELL^®^ Advanced Hand Sanitizer Green Certified Gel. Additional information on test dispensers A-D (including photographs) and test ABHS formulas used in accelerated stability studies are found in S1 File.

Each of the four test dispenser types were filled with 1L of each of eight ABHS types (products A through H), resulting in a total of 32 dispenser × ABHS combinations. Filled dispensers were then stored in environmental chambers (Bahnson Environmental Specialties Model LLC/ES2000, Raleigh, NC) at 40 ± 2°C, RH of 75 ± 5% for six months, which simulates 36 months of storage at ambient conditions using an Arrhenius extrapolation. These conditions are based on “accelerated” storage conditions in accordance with drug stability testing guidance issued by the FDA [18]. During accelerated storage, duplicate aliquots of ABHS were aseptically collected at 0, 1, 2, 3, and 6 months for alcohol content analysis. Data obtained from the combination of dispenser D with ABHS E was not available for ethanol analysis due to visible leaking during storage.

### Statistical analysis

Microsoft Excel (Microsoft, Redmond WA) was used for preparation of all tables, graphs, and charts, as well as performing one way ANOVA tests. Individual ANOVA tests were performed exploring dispenser type, dispenser cap status, and ABHS type as treatments, with ethanol loss as the independent variable. Tukey’s HSD post-hoc test was used for separation of means after analysis. Statistically significant associations were considered *P <* 0.05. Averages are presented in results, and error bars in all Figs represented standard error of the mean.

## Results

### Quality attributes and product labeling

A summary of the quality characteristics and the consistency of product labeling with the FDA guidance document (from available photographs) are presented in Table 1 for the 117 samples collected in this study. All 40 ABHS samples collected from the school district were colorless and transparent (Table 1). For the 77 ABHS samples collected from community settings, over 93% of the samples were colorless (n = 72/77) and over 85% of the samples were transparent (not cloudy or opaque) (n = 66/77). The only observed colored hand sanitizers were green (n = 4) and yellow (n = 1), with color apparent throughout the entire solution, and 14.3% (11/77) of samples contained at least one visual impurity. Most visual impurities observed were in the form of white particulates in the sanitizer solution (n = 7), with some sanitizer samples appearing cloudy (n = 1), containing black (n = 1) or black and brown (n = 1) particulates. Notably, one bulk refillable dispenser that was shipped to GOJO Industries had visible evidence of mold growth on the outside of the container.

**Table 1:** Visual and quality characteristics of ABHS samples obtained from bulk refillable dispensers in community settings.

Location	Color		Appearance ^a^		Full Product Labeling ^b^	
School District (n = 40)	Colorless	100.00% (40/40)	Transparent	100.00% (40/40)	No	100.00% (40/40)
Community Settings (n = 77)	Colorless	93.50% (72/77)	Transparent	85.71% (66/77)	No	50.87% (29/57)
			White particulates in solution	9.09% (7/77)		
	Green	5.19% (4/77)	Mold observed on dispenser	1.30% (1/77)	Unknown	40.35% (23/57)
			Cloudy solution	1.30% (1/77)		
	Yellow	1.30% (1/77)	Black particulates in solution	1.30% (1/77)	Yes	8.77% (5/57)
			Black and brown particulates in solution	1.30% (1/77)		

Summary of visual, quality, and labeling characteristics of 117 ABHS samples taken from a single school district (n = 40) or from bulk refillable dispensers in community settings (n = 77).

^a^ Visual appearance for each sample was assessed through trained laboratory personnel visually evaluating each sample compared to a reference lot of PURELL^®^ Advanced Gel (GOJO Industries, Akron, OH). Samples with no obvious visual impurities (e.g., transparent, homogeneous sample with no turbidity) are marked “transparent”. Samples with visible impurities are noted with a brief description of each impurity. Particulates are defined as large visible particles that settle to the bottom of the sample cup after shaken).

^b^ Sample collectors were asked to take photos of each dispenser at the time of sample collection. Upon observation, status of product labeling for each dispenser was categorized into one of the following categories: 1) No: Adequate photos were taken and one or more of the following items were missing from the dispenser and/or refillable reservoir: Drug Facts Label, manufacturer and product identifier, lot code, and expiration date; 2) Unknown: photos taken, but not enough to draw a full conclusion (e.g., locked outer dispenser with no access to refillable reservoir); 3) Yes: Adequate photos were taken and Drug Facts Label, product and manufacturer identifier, lot code, and expiration dates are visible and appear to be acceptable. Note that photographs from 20 dispensers from community settings were not available are thus excluded from this column.

Using photos collected by the trained sample collectors, dispensers from the field were also evaluated for the presence of crucial information on the product label, including the Drug Facts Label (required by FDA for OTC Drug manufacturers), manufacturer and product identifier, lot code and expiration date (Table 1). Of the 40 ABHS samples collected from the school district, not a single dispenser had adequate product labeling (Table 1). Of the 57 samples collected from community settings where photos were available, 50.87% (29/57) of dispensers lacked one of these key pieces of label information and were considered to not have full product labeling. Only 8.77% (5/57) of dispensers where sufficient photographs were available had full product labeling (i.e., containing the Drug Facts Label, manufacturer and product identifier, lot code, and expiration date).

### FDA impurity analysis

A summary of results for FDA impurities (i.e., methanol, benzene, acetal, and acetaldehyde) are shown in Table 2. Due to the destructive nature of the alcohol concentration analysis method (which preceded the FDA impurity analysis), and the fact that some samples had to be analyzed multiple times, a total of 26 samples collected from community settings did not have sufficient material for FDA impurity analysis after alcohol analysis was completed. This resulted in a total of 91 samples being analyzed for FDA impurities (51 from community settings and 40 from the school district). The vast majority of FDA impurities were observed in ABHS samples from community settings; only a single sample in the school district showed presence of acetal at excessive levels (511 ppm; Table 2). In ABHS samples from community settings, acetal (35.29%; 18/51) and acetaldehyde (33.33%; 17/51) were the most frequently identified chemical impurities in excess of FDA’s Guidance Document [10]. Methanol in excess of FDA’s interim limits was observed in 5.88% (3/51) of community acquired samples, and benzene was not detected in any the 91 samples analyzed. Average analyte content of methanol, acetal, and acetaldehyde from non-compliant samples acquired from community settings were 1,626, 271, and 202 ppm, respectively. All 91 ABHS samples were also analyzed for an additional 8 impurities (e.g., acetone, n-propanol, etc.) as indicated in FDA’s Guidance Document [10]. While the sum of all 8 of these impurities exceeded 300 ppm for several samples, upon further investigation, all samples contained chemical impurities under the interim limits for each analyte and considered compliant (S1 Table).

**Table 2:** Results of FDA impurity analysis of ABHS samples from bulk refillable dispensers.

Location	Analyte (Interim Threshold) ^a^	Samples Non-Compliant with Temporary Guidance Document ^b^	Average Content of Non-Compliant Samples (ppm)
Community settings (n = 51)	Methanol (630 ppm)	5.88% (3/51)	1,626 (± 610)
	Benzene (2 ppm)	0.00% (0/51)	N/A
	Acetal (50 ppm)	35.29% (18/51)	271 (± 227)
	Acetaldehyde (50 ppm)	33.33% (17/51)	202 (± 160)
School district (n = 40)	Methanol (630 ppm)	0.00% (0/40)	N/A
	Benzene (2 ppm)	0.00% (0/40)	N/A
	Acetal (50 ppm)	2.50% (1/40)	511
	Acetaldehyde (50 ppm)	0.00% (0/40)	N/A

Summary of FDA chemical impurity testing for ABHS samples collected from bulk refillable dispenser in community settings (n = 51) and from a school district (n = 40).

^a^ Interim thresholds established by FDA’s Temporary Policy for Preparation of Certain Alcohol-Based Hand Sanitizer Products During the Public Health Emergency (COVID-19)—Guidance for Industry [10] are listed in parentheses. Samples in excess of these thresholds are considered non-compliant.

^b^ Non-Compliant products are those where the analyte content was observed to be in excess of thresholds established by the Guidance Document [10]. Of the 117 total samples collected, 26 samples from community settings did not have sufficient material (after alcohol analysis) for FDA impurity analysis, yielding a total of 51 samples analyzed for these impurities. All 40 samples from the school district contained sufficient material for analysis. Standard deviation is provided in parentheses where averages are reported.

### Alcohol concentration analysis of ABHS dispensers

A summary of the alcohol concentration analyses appears in Table 3. A total of 33.62% (39/116) of samples collected were found to have low concentrations of alcohol (defined as under 60%), which are non-compliant with FDA OTC drug regulation for topical antimicrobial drug products (i.e., the active ingredient is too low). Most non-compliant samples were from the school district (82.05%; 32/39). A total of 9.09% (7/77) of ABHS samples from community settings were non-compliant. All non-compliant samples contained ethanol as the active ingredient (no products formulated with isopropanol as the sole active ingredient were found to be non-compliant). The average ethanol concentration of these non-compliant samples was 44.57%, while the range for all samples were 16.21 to 87.44% (Table 3).

**Table 3:** Results of alcohol concentration analysis of ABHS samples from bulk refillable dispensers.

Location	Number with Non-Compliant Alcohol Concentration ^a^	Average Alcohol Concentration of Non-Compliant Samples ^c^	Range of Ethanol Concentration of All Samples
Community settings (n = 77)	9.09% (7/77)	43.55% (±12.92%)	24.69 to 87.44%
School district (n = 39)	82.05% (32/39 ^b^)	44.79% (±9.61%)	16.21 to 71.84%

Subpotent and non-compliant alcohol concentrations found in ABHS samples from bulk refillable dispensers in community settings and from a single school district.

^a^ Non-Compliant is defined as having less than 60% (v/v) ethanol, or less than 70% (v/v) isopropyl alcohol, as indicated by US FDA regulations governing the sale of hand sanitizers [23]. No ABHS samples with isopropyl alcohol as the sole active ingredient were found to be non-compliant.

^b^ One of the samples collected from the school district did not have enough material for a re-run after alcohol analysis failure and was excluded from the results.

^c^ Concentration is reported on a volume basis. Standard deviation is provided in parentheses where averages are reported.

**Fig 2:**
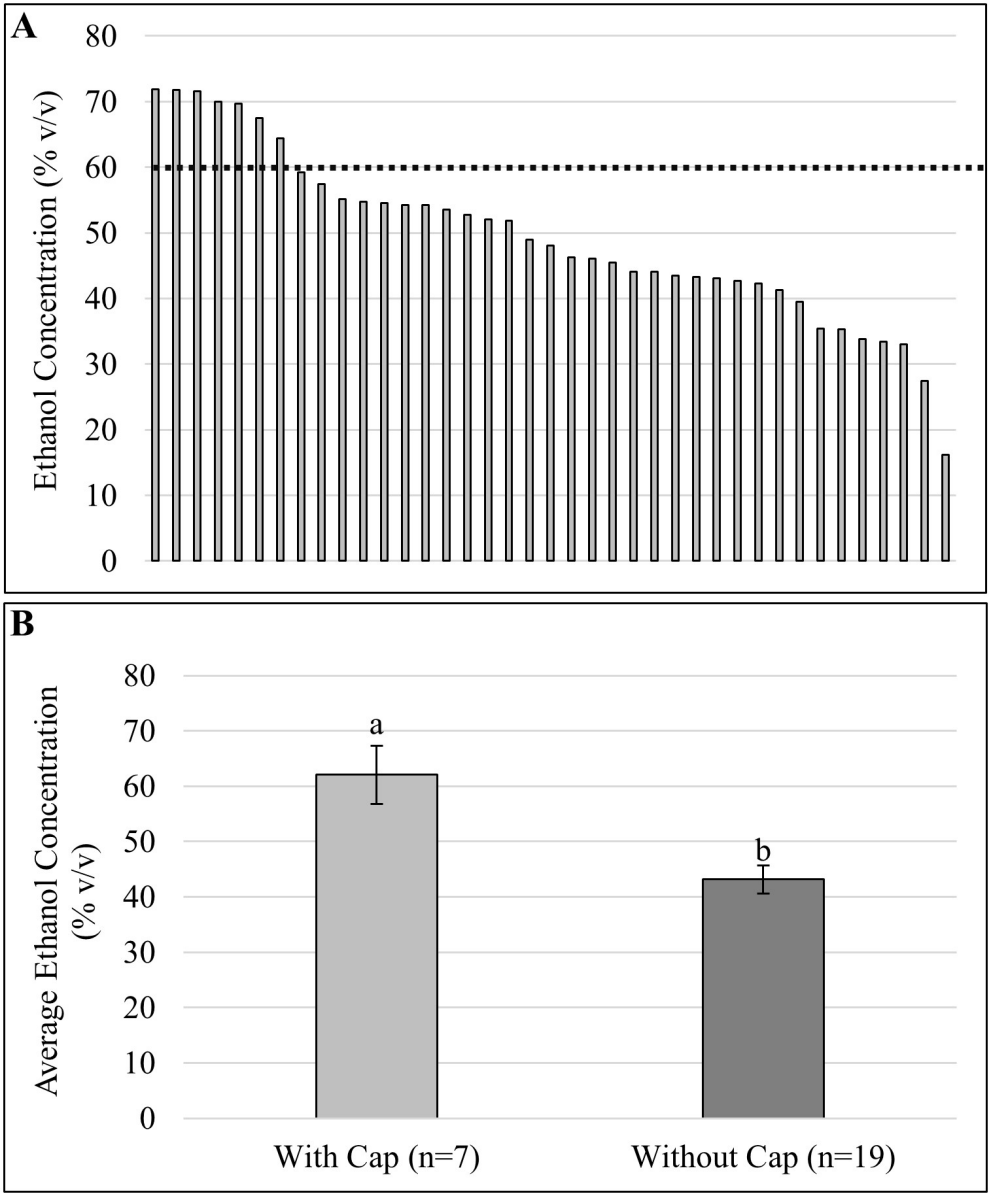
Ethanol concentration in ABHS samples sourced from single school district. Ethanol concentration of ABHS samples from single school district in South Carolina. Panel A: Distribution of ethanol concentrations (% v/v) measured from 39 individual dispensers. Black dashed line indicates 60% ethanol, the minimum concentration of ethanol required for the ABHS product to be compliant with FDA OTC Drug regulations [23]. Panel B: Average ethanol concentration is associated with status of refill cap placement. A total of 39 ABHS samples from a single school district was analyzed for ethanol concentration. Upon inspection of photos, many dispensers had refill caps removed (n = 19), some had caps installed (n = 7), and some were unable to determine based on photographs (n = 13). When grouped by status of cap placement, a significant difference in average ethanol concentrations (% v/v) was observed. Refill cap status (with or without) was significantly associated with rate of ethanol loss (*P* < .05) based on one-way ANOVA with cap status as treatment and average ethanol concentration as independent variable. Letters above each bar graph indicate statistically different mean ethanol percentages using Tukey’s HSD post-hoc test for separation of means.

As shown in Table 3, most non-compliant ABHS samples were from a single school district. After reviewing dispenser photographs from this school district, it was determined that all 39 samples available for alcohol content analysis utilized the same dispenser design: a refillable manual dispenser with a secured refill cap not intended to be removed during use. Evaluation of photographs available from the school district samples show that 19 of the dispensers had caps removed, presumably to promote ease of refilling. Seven dispensers had the caps installed, and cap install status could not be determined from the remaining 13 photographs. Ethanol concentration results of these 39 dispensers are presented in Fig 2. When caps were present, ABHS samples sourced from dispensers were shown to have an average ethanol content of 62.06%, while ABHS samples from dispensers without caps present were shown to have an average ethanol content of 43.14%, a statistically significantly lower level (*P* < 0.05). Given that this district used only one ABHS bulk product at the time of sample collection, these results suggest tampering and improper use of the refill cap on the dispenser internal reservoir led to faster evaporation of ethanol content.

### Impact of dispenser and ABHS type on rate of ethanol evaporation

**Fig 3:**
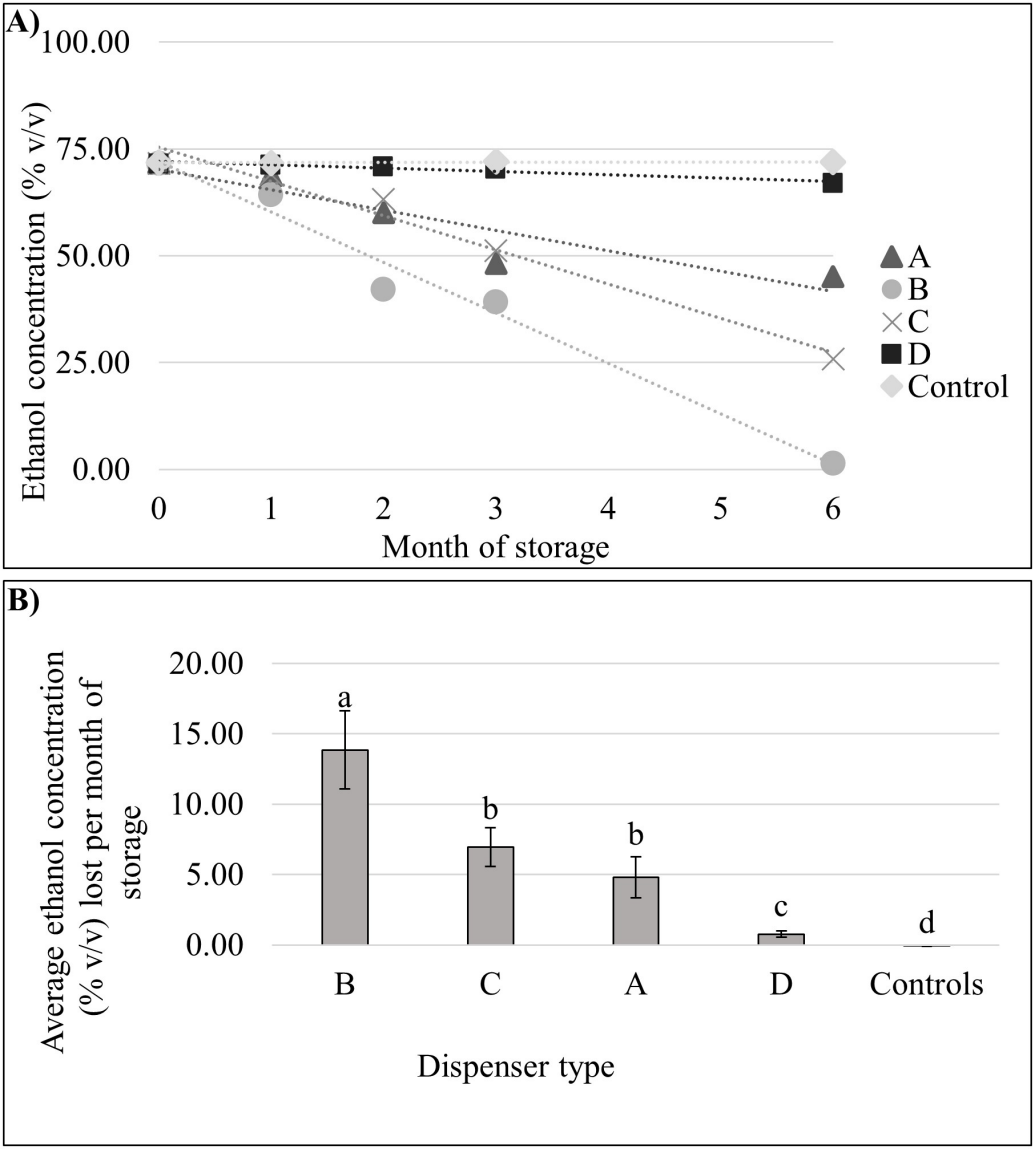
Observed ethanol loss in ABHS stored in bulk refillable dispensers (pooled across all ABHS types) under accelerated stability conditions (40 ± 2°C, RH of 75 ± 5%). Rate of monthly ethanol loss of ABHS stored in various bulk refillable dispensers held in accelerated stability conditions. For both panels, ethanol content is pooled across all ABHS types (e.g., foam, gel, liquid/WHO), since ABHS type was not found to significantly impact the rate of ethanol evaporation (data shown in S1 Fig; *P* > 0.05). Controls were two sanitary sealed 1200mL refill bottles of ABHS products from GOJO Inc. (details in Materials and Methods). Panel A: Observed ethanol loss over 6 months of storage time for ABHS stored in bulk refillable dispensers. Samples were stored at 40 ± 2°C, RH of 75 ± 5%, and aliquots for analysis were measured after 0, 1, 2, 3, and 6 months of storage. Bolded shapes on the graph represent mean ethanol percent (v/v) values for duplicate aliquots at each indicated time point. Dashed lines represent a linear fit trendline based on all data points. Dispenser types appear on the legend, and details of each dispenser type appear in S1 File in the supplemental materials. Panel B: Monthly loss of ethanol represented as the inverse slope of a linear trendline of ethanol content as a function of time when dispenser × ABHS combinations were stored for 6 months at accelerated conditions (from Panel A). Error bars represent standard error of the mean. Dispenser type was significantly associated with rate of ethanol loss (*P* < .05) based on one-way ANOVA with dispenser type as treatment and average monthly ethanol loss as independent variable. Letters above each bar graph indicate statistically different mean ethanol percentages using Tukey’s HSD post-hoc test for separation of means.

To better understand the impact of bulk refillable dispenser design as well as ABHS format on ethanol evaporation rates, we performed a laboratory experiment by filling various commercial dispensers (A-D) with multiple ABHS products representing three sanitizer formats (gel, foam, WHO [liquid] formulation) and stored them at accelerated stability conditions (40 ± 2°C, RH of 75 ± 5%) for six months. These conditions are based on guidelines outlined by the FDA [18] and have been previously determined to approximate 36 months of storage at ambient conditions. As shown in Fig 3, dispenser type was statistically associated with rate of ethanol loss (*P* < 0.05). The sanitary-sealed refills used as controls (depicted by grey diamond) performed the best, with minimal loss of ethanol concentration over the entire 6 months of storage. The worst performers were dispenser types that lacked a sealed internal reservoir. Specifically, dispensers B (depicted by grey circle) and C (depicted by grey X), at each month lost on average 13.86% and 6.95% ethanol concentration by volume, respectively. Dispensers with a sealable internal reservoir performed better, with dispenser D each month losing on average 0.77% ethanol concentration by volume. ABHS format was not statistically associated with ethanol loss (S1 Fig; *P* > 0.05).

## Discussion

Demand for ABHS has been elevated throughout the COVID-19 pandemic, which has triggered product shortages. To alleviate these shortages, many government agencies that regulate ABHS have instituted temporary policies allowing for use of lower quality alcohols as an active ingredient for ABHS [8–10]. Unfortunately, this has led to several reports of inferior, and poor quality, ABHS products entering the market with high levels of chemical impurities [13–15,21]. To our knowledge, only one study has examined the frequency at which these inferior products are offered for public use in community settings [21]. Additionally, we are not aware of any studies to date that have been performed to further characterize risks associated with use of ABHS in bulk refillable dispensers. To fill these knowledge gaps, ABHS samples were collected from bulk refillable dispensers in real world settings across the United States and their product quality and safety profiles were assessed. Additionally, the alcohol content of these samples was examined, and then the impact of dispenser design on ethanol evaporation rates in laboratory-based experiments was determined. Collectively, the results highlighted in this study show risks associated with use of some ABHS and bulk refilling practices prevalent during the COVID-19 pandemic.

### Bulk refillable dispensers lack critical product labeling

In this study, 71.13% (69/97) of all dispensers with photos available lacked adequate product labeling (Table 1). For these dispensers, at least one of the following was missing from the primary dispenser: FDA required “Drug Facts Label”, manufacturer and product identifier, lot code, and expiration date. These labeling items are critical for communicating key messages to the consumer. The Drug Facts Label, which is required by law for all FDA regulated OTC drug products, communicates key information, such as the active and inactive ingredients of the product, the purpose and use indications for the product, how to use and store the product, and any warnings that may be associated with the product [22]. By using ABHS from a bulk dispenser without a Drug Facts Label readily visible on the primary package, consumers may incorrectly use the product, or may inadvertently use a product formulated with an inactive ingredient that is incompatible or allergenic with their skin.

The lack of lot codes is also a major concern due to the inability to trace a product in the event of a recall. This is especially concerning given that the FDA’s “Do Not Use List” represents over 250 products [13]. The issue of traceability is further complicated by the ease at which multiple products can be mixed within a bulk refillable dispenser, leading to a situation where it is not clear what exact product is in the dispenser, making traceability likely impossible in such a scenario.

Finally, the lack of an expiration date on most samples is also problematic. Since these non-traditional manufacturers would not have processes and procedures to assess product stability and shelf life, without a printed expiration date, these products could remain on the market for long periods of time past their intended shelf life, which puts their safety and efficacy profile at risk. Collectively, the high observations of non-compliant OTC Drug Labeling on bulk refillable ABHS dispensers warrant regulatory action and clarification.

### Evidence of visible product defects and dual active ingredients in ABHS samples may indicate mixing of multiple products

While no ABHS samples from the school district contained visible defects, we observed visible defects in 14.3% (11/77) of ABHS samples obtained from community settings, with the majority being particulates in solution (Table 1). These particulates indicate the presence of insoluble materials in solution. It is not known whether these observed visible defects are a result of issues during ABHS manufacture or from mixing products within the bulk refillable dispenser. The presence of visible impurities could result from mixing, especially if two incompatible ABHS formulas were used that promote production of unwanted by-products. ABHS formulas differ substantially in their formulaic composition, and may include a variety of gelling agents, pH ranges, and salt content. Long term stability of an ABHS formula is often dependent on these compositional considerations. Mixing of two formulas with vastly different stability characteristics could promote the production of unwanted by-products, for example the precipitation of gelling agents due to a shift in pH and/or salt content. The observed visual impurities could also indicate incompatibility of ABHS products with packaging components or incompatibility of ABHS products with components of the dispensing systems. It is worth noting that at least one of the samples with visible impurities had evidence of dual active ingredients, with ethanol and isopropanol levels measured at over 39 and 29% by volume, respectively. We observed a further three ABHS samples to have both ethanol and isopropanol in appreciable amounts (i.e., over 15% by volume), further lending support for evidence of mixing of multiple products in other bulk refillable dispensers (S1 Table). The act of mixing different formulas carries risk as it can result in an unsafe or a non-efficacious chemistry. These mixtures are also out of compliance with FDA regulations governing the sale of OTC hand antiseptic products, which do not allow multiple active ingredients within a single commercial formulation [23].

At least one dispenser appeared to have previously been used as a bulk refillable dispenser for soap and had evidence of mold at the output valve of the dispenser. Bulk soap dispensers can become a public health risk if not adequately maintained from a sanitary perspective. Research has shown that bulk soap dispensers can become contaminated with bacterial biofilms and bacterial pathogens, and that transfer of bacteria from the dispenser to hands can readily occur during handwashing [16,24]. It is not clear if a contaminated bulk soap dispenser repurposed for dispensing ABHS is a public health risk, but at the very least, the dispenser should be thoroughly cleaned and sanitized prior to repurposing for use with other products.

### ABHS samples from bulk dispensers frequently contain chemical impurities over FDA interim limits, which may pose health risks

In 91 ABHS samples analyzed for FDA impurities, we observed samples non-compliant (i.e., containing over FDA interim production limits) for methanol, acetaldehyde, and acetal presence (Table 2). Nearly all impurities identified were from ABHS samples sourced from community settings, rather than the single school district. This seems logical as the school district was using a single ABHS product. A total of 5.88% (3/51) of community acquired samples were found to be non-compliant for methanol, with the average methanol content of these samples over 2.5 times higher than FDA’s interim limit of 630 ppm (average methanol content of non-compliant samples was 1,626 ppm; Table 2). Other studies examining ABHS used in community settings have found widespread methanol contamination. For example, in an analysis of 265 samples of ABHS used in community settings in Malaysia during 2021, 18% of samples had detectable methanol levels, and 3 samples had methanol concentrations of at least 40% (v/v) in the ABHS formulation [21].

Methanol, a natural by-product of fermentation, can become present in an ABHS formulation through improper distilling or other inadequate purification methods [25]. Methanol can be absorbed via ingestion, inhalation, or excessive skin contact, and is ultimately metabolized by the liver. While toxic side effects related to dermal absorption of methanol contaminated ABHS are rare, there have been documented cases in individuals in healthcare settings who use ABHS frequently. In one example, six surgeons in a Chinese hospital developed extreme erythema after frequent use of an ABHS contaminated with methanol at 3,000–5,000 times the legal limit [26]. One surgeon developed neurological issues and blurred vision but recovered once use of the contaminated ABHS ceased. Additionally, excess methanol ingestion can lead to formation of formic acid, the primary toxic metabolite responsible for organ damage, and death can occur if excessive amounts are ingested within a short amount of time [26].

Excess (greater than 50 ppm) levels of acetaldehyde and acetal were observed in 33.33% (17/51) and 35.29% (18/51) of ABHS samples acquired from community settings, respectively (Table 2). Only 1 ABHS sample acquired from the school district was in excess of 50ppm for acetal. Acetaldehyde and acetal were the most frequently encountered chemical impurities above FDA interim limits in the current study. Other studies performing analytical evaluations of commercially available ABHS products have shown acetaldehyde and acetal to frequently be the most encountered chemical impurity. For example, in 2021, researchers analyzed 42 commercially available ABHS products for nine common impurities to determine compliance with Health Canada interim production guidelines [15]. Similar to the current study, these researchers found 26.2% (11/42) of samples analyzed had levels of acetaldehyde in excess of 75 ppm, the Health Canada interim guidance threshold. Another study in 2021 examined ethanol content and chemical impurities of 48 commercially available ABHS products in Brazil, finding that 25% (12/48) had high amounts of acetaldehyde in excess of the Brazilian Health Regulatory Agency’s guidelines for interim production [27].

Acetaldehyde is produced through an oxidation reaction of ethanol by alcohol dehydrogenase and is readily converted between acetal in the presence of ethanol and an acid catalyst [28]. Inhalation is the primary route of exposure of acetaldehyde, but dermal absorption can also occur. High levels of acute exposure can cause severe lung irritation, while prolonged exposure can lead to carcinogenic effects, as acetaldehyde is recognized as a likely carcinogen [28] and is categorized as Group 2B classification by the International Agency for Research on Cancer. Collectively, the results presented in the current study and others suggest a wide range of ethanol suppliers currently used globally in manufacture of ABHS products under temporary guidance policies may have excessive levels of acetaldehyde and/or acetal. More vigilance is needed by local regulatory authorities in these areas to avoid these potentially unsafe ABHS products entering the market, which may pose health risks with excessive use.

Benzene was not detected in any of the 91 samples analyzed for chemical impurities (Table 2). While several other analyses of commercially available ABHS products failed to show any samples with appreciable levels of benzene [15,27], benzene has been previously shown to be a concern in commercially available ABHS products for sale to consumers in retail stores in the United States [14], with 8.07% (21/260) of these samples having benzene levels above 2 ppm, the interim threshold set by the US FDA. Exposure to benzene has long been associated with health risks, as it is considered a known carcinogen and can be absorbed through skin and inhalation [29]. Severe symptoms from prolonged exposure include anemia from loss of blood cells, which can lead to death.

In FDA’s emergency guidance, FDA stipulates that the guidance and therefore the relaxed impurities limits, only applies to liquid ABHS and not gel based or aerosol ABHS. Non-emergency limits for some contaminants like benzene are not well defined for ABHS other than FDA guidance stated that benzene is classified as a “Class 1 solvent” that “should not be employed in the manufacture of drug substances, excipients, and drug products because of their unacceptable toxicity” [30]. Regulators should clarify limits for these contaminants and chemical impurities in non-emergency settings for all ABHS products, regardless of format.

### ABHS samples frequently had low concentrations of ethanol, which is linked to improper use of dispensers and/or dispenser design

We evaluated the alcohol content (both ethanol and isopropanol, two active ingredients allowed by FDA’s interim ABHS production guidelines) for 116 total ABHS samples, 39 from a single school district and 77 from community settings. Over 82.05% (32/39) of ABHS samples collected from bulk refillable dispensers in the single school district had ethanol levels under 60% by volume (Table 3). For ABHS samples collected from bulk refillable dispensers in community settings, 9.09% (7/77) had ethanol levels under 60% by volume (Table 3). Sixty percent is the minimum alcohol concentration by volume required by the US FDA in ABHS products; any ABHS sold with an ethanol content under this amount is non-compliant according to federal OTC Drug manufacture regulations [23]. The average ethanol content of all non-compliant samples was 44.57% by volume, while the lowest observed ethanol content of any sample was 16.21% by volume (Table 3). No samples with isopropanol as the sole active ingredient were found to have non-compliant levels.

Several studies examining the alcohol content of ABHS during the COVID-19 pandemic have been published. For example, a 2021 analysis of 42 commercially available ABHS products in Canada identified 2.4% (1/42) of ABHS products having an ethanol content below 60% (v/v) [15]. In another study of commercially available ABHS products in Brazil, 4.2% (2/48) of samples were found to have an ethanol content below 70% (v/v) [27]. Finally, in an analysis of 386 ABHS samples in Malaysia, researchers found 17.4% of commercially available ABHS samples had an ethanol content below 60% (v/v) [21]. These researchers also found 42.3% of ABHS samples taken from publicly available dispensers in community settings had an ethanol content below 60% (v/v) [21]. To our knowledge, this is the only previously published study specifically focused on examining community placed dispensers, although the researchers focused on both bulk dispensers as well as bottles.

One difference in the current study and these previous studies is that we focused exclusively on collecting ABHS samples from bulk refillable dispensers in community settings, which are suspected to be associated with evaporation [17]. This, along with the laboratory-based ethanol evaporation data presented elsewhere in this manuscript, support the notion that the practice of bulk refilling ABHS dispensers may contribute to rapid evaporation of alcohols, such as ethanol, which may render the ABHS ineffective.

Upon closer review of the photos and the ethanol results from the single school district in SC, an association between placement of the dispenser’s refillable reservoir cap and average ethanol content of ABHS samples was observed (Fig 2). Each dispenser from this school district was of the same design: a manual ABHS dispenser with an internal reservoir containing a tightly sealed plastic cap, designed to facilitate rapid refilling by a centrally located dispenser while minimizing evaporation in between fillings. In 19 of the 39 samples analyzed, the refill caps were physically not present on the dispenser reservoir at the time of sampling. We found that removal of the cap was statistically associated with observed lower ethanol percentages of ABHS samples from this school district (*P* < 0.05; Fig 2), indicating that improper use of the dispenser (i.e., physical removal and/or not replacing the cap) may have resulted in faster evaporation of ethanol. Currently, controlled experiments using the same combination of dispenser and ABHS product are ongoing to test this hypothesis.

To further evaluate the impact of bulk refillable dispenser design and ABHS format on ethanol evaporation rates, we devised a series of laboratory experiments utilizing accelerated drug stability test conditions using bulk ABHS product and dispensers purchased from e-commerce sites (Fig 3). In these experiments, we filled various ABHS dispensers with several commercially available ethanol based ABHS products and stored them in environmental chambers for six months at 40 ± 2°C (RH of 75 ± 5%). Based on previous internal data obtained, these conditions approximate 36 months of storage at ambient conditions. Our results show a significant effect of dispenser type on ethanol evaporation rate (*P* < 0.05; Fig 3). There was no significant effect of ABHS format type (e.g., gel, foam, WHO [liquid]) on ethanol evaporation rate (S1 Fig; *P* > 0.05).

Bulk refillable dispensers without an internal reservoir had the highest rates of ethanol loss over time (dispenser types B and C, Fig 3). Dispenser type B, which was a bulk soap style dispenser of plastic construction with an open refillable basin, had a significantly higher rate of ethanol evaporation as compared to other dispensers tested (*P <* 0.05), as it lost an average of 13.86% (v/v) of ethanol on a monthly basis (Fig 3B, dispenser B). After two months of storage, the average ethanol content of ABHS samples stored in Dispenser type B was 42.15% (v/v), which is under the FDA required minimum content of 60% (v/v) for ABHS sold in the United States. After six months of storage, the average ethanol content of ABHS samples stored in Dispenser type B dropped even further to 1.4% (v/v) (Fig 3A). Dispensers A and D, which both contained an internal refillable reservoir with a cap, performed significantly better than the open basin style dispensers (*P <* 0.05; Fig 3). Dispenser D performed the best out of all test dispensers, losing an average of 0.77% (v/v) of ethanol on a monthly basis (Fig 3B, dispenser D). The two control dispenser refills significantly outperformed all other test products and showed no appreciable loss of ethanol content over time (Fig 3). These results confirm that sealed dispenser refills are best for preventing ethanol loss during storage, and that refillable dispensers with internal reservoirs reduce ethanol evaporation rates as compared to refillable dispensers without an internal reservoir. Companies, organizations, and individuals wishing to adopt bulk refillable ABHS should be aware of these significant evaporation risks associated with dispenser design. Some dispenser designs, such as bulk refillable dispensers with an open refillable cavity designed for soap use, should not be used with ABHS products, as rapid evaporation of alcohol readily occurs and may results in an ineffective product.

Alcohol-based active ingredients, such as ethanol and isopropanol, are critical for efficacy of hand sanitizers against pathogenic organisms, such as SARS-CoV-2 [31,32]. In bacteria, these active ingredients achieve their antimicrobial effect by disruption of key metabolic pathways, induce irreversible damage to cellular membranes, and ultimately, complete loss of cellular integrity [32]. The mechanisms of alcohol inactivation of viruses are less understood but are thought to disrupt lipid bilayers in enveloped viruses and disrupt cellular receptor binding sites in non-enveloped viruses [32]. The percent of alcohol within an ABHS formulation is critical for efficacy, and it is generally thought that a minimum of 60% alcohol (v/v) is required for an efficacious product [32]. Research on SARS-CoV-2 has shown that a minimum of 30% was required for efficacy, though the authors recommend prioritizing formulas with 60% or greater [31]. Sixty percent (v/v) ethanol is also the minimum amount required by the US FDA for ethanol-based ABHS products sold in the United States [23]. Use of ABHS with sub-potent levels of alcohol by consumers is a health risk, as an ineffective product could result in an increased risk of infection. To reduce the chances of ABHS evaporation in community settings, the use of open refillable dispensers, especially those originally designed for dispensing soap through an open refillable cavity without a sealed reservoir, should be discouraged.

### Research implications for regulators

The high rate of non-compliant ABHS products (which may pose public health risks) observed in community settings, as well as the link between ethanol evaporation and use of ABHS in bulk refillable dispensers, have major implications for regulatory agencies that oversee the sale and manufacture of these products. These agencies should consider actions to minimize risks associated with use of these inferior products and practices.

First, it could be argued that temporary guidance policies allowing for non-traditional ABHS manufacturers to quickly enter the market using low quality ethanol are partly responsible for the flood of inferior products and practices entering the market. Where possible, agencies who oversee these policies should consider withdrawing these policies, as ABHS supply from major manufacturers has returned to pre-pandemic levels. In fact, the US FDA recently has announced the withdrawal of their temporary policy, set to expire on December 31, 2021 [11], attributing the withdrawal to the increased availability of ABHS in the market. If agencies are unable or unwilling to withdraw these policies, ongoing surveillance is required to ensure inferior products are proactively identified and recalled when necessary.

Second, regulators should provide clarity and issue guidance on the practice of bulk refilling and dispensing of ABHS (an OTC drug product), due to the risks of ethanol evaporation, product mixing, and lack of required labeling on the primary package. Development and release of a guidance document for industry on best practices would be beneficial to provide clarity and minimize risks. Regulators should also clarify limits for chemical contaminants and chemical impurities in non-emergency settings, as they are not clearly defined for OTC drug products.

Finally, as temporary policies for ABHS production are withdrawn, consumers and professionals alike are left to deal with disposal of ABHS that may be expired and/or of inferior quality. Because of its flammability, ABHS is considered a hazardous material in many areas, which makes disposal complicated and confusing, especially for large amounts of product. A guidance document outlining best practices for disposal of these ABHS products would be extremely beneficial to the general public and the industry as a whole and would ensure these products are disposed of properly so that any risks during disposal are minimized. To facilitate ease of disposal by the general public, national drug collection sites managed by the US Drug Enforcement Administration could potentially be explored as a venue for disposal of these ABHS products.

### Research limitations and future research needs

While the research presented here is valuable, we acknowledge some limitations of our study design. First, because dispensers in community settings were sampled during a single point in time, our study design cannot account for the conditions that dispensers were exposed to prior to the point of sampling. For example, adverse weather effects, such as excessively hot periods of time, could impact rate of ethanol evaporation of ABHS in refillable dispensers prior to our sampling. Inadequate use of dispensers (e.g., refillable top left open for long periods of time), could also impact rate of ethanol evaporation. Finally, the ABHS formulation itself could potentially impact the evaporation rate of alcohol, given that isopropanol has a lower vapor pressure (4.40 kPa at 20°C) as compared to ethanol (5.95 kPa at 20°C). A future controlled evaporation study investigating the impact of choice of active ingredient (i.e., ethanol versus isopropanol) is warranted. While this study was not designed to account for the above factors that could potentially impact evaporation rate, it still holds value as ABHS samples collected were accessible for use by the general public in community settings.

Another limitation of our study is that we cannot determine whether the issue of chemical impurities (e.g., methanol, acetaldehyde/acetal) is related to the production of ABHS, the use of bulk refillable dispensers (e.g., mixing multiple products), or some combination of both. It is worth noting that these contaminants are often by-products of ethanol production and purification but could also be from other raw materials. Future studies sampling both bulk refillable dispensers and their associated ABHS products from unopened refills would help to determine the key factors contributing to the high levels of chemical impurities observed in ABHS products.

While this study clearly demonstrates a link between use of bulk refillable dispensers and elevated ethanol evaporation rates in ABHS stored in these dispensers, it is not yet known what exact impact this evaporation has on antimicrobial efficacy. Future studies looking more closely at the relationship between dispenser type, evaporation of ethanol, and pathogen reduction of ABHS using both *in vitro* and *in vivo* methods would be helpful.

Finally, one area not addressed in this study was sampling of ABHS from individual bottles that are refilled. It is not known if ethanol evaporation is associated with these individual bottles, although refilling of individual bottles is likely associated with risks from mixing and lack of compliant labeling information. Future studies are warranted that examine the risks of refilling individual bottles relative to bulk refillable dispensers.

## Conclusions

In this study, we sought to better characterize the risks associated with ABHS products of unproven quality and bulk refilling practices of these products during the COVID-19 pandemic. To do this, we collected samples from “real world” community settings and assessed the product quality and safety profile of these samples. Additionally, we analyzed alcohol concentration of ABHS stored in bulk refillable dispensers, and then determined the impact of dispenser type on ABHS evaporation rates using laboratory experiments. Collectively, the data presented in this study show that ABHS from community settings:

1. Frequently contain visual impurities and lack appropriate required consumer facing labeling, which can mislead individuals on use instructions and product risk profiles.
2. Can contain high levels of potentially harmful chemical impurities in excess of interim federal guidance, resulting in a product that is potentially harmful if misused.
3. Frequently contain sub-potent levels of alcohol, which may result in an ineffective product that puts individuals at unnecessary risk.
4. Are associated with higher rates of alcohol evaporation due to dispenser design and misuse, especially when bulk refillable dispensers are used.

This study illustrates why individuals should be diligent when choosing an ABHS product and manufacturer. All ABHS manufacturers, even those operating under FDA’s interim guidance policies, are manufacturing Over-The-Counter Drug products. Strict FDA regulations require these products to be produced in a safe manner and concerned users of ABHS should not hesitate to ask ABHS manufacturers for proof of adherence to these regulations. Adoption of inferior ABHS products and practices puts individuals at unnecessary risk, especially in settings housing vulnerable populations (e.g., daycares, K-12 schools, hospitals, long-term care facilities). Individuals and organizations wishing to adopt use of ABHS dispensers should also prioritize use of sanitary sealed refills, as they are not associated with loss of alcohol content over time. Regulatory agencies globally who are currently overseeing interim guidance allowing for temporary production of ABHS with relaxed requirements, should consider whether the guidance is needed and should consider strengthening all hand hygiene guidance and regulations, as there may be unintended public health risks, both to human safety and to infection prevention, associated with these policies and practices.

## Supporting information

S1 Fig Impact of ABHS format type on evaporation rates. (TIF)

S1 Table Summary of samples. (XLSX)

S1 File Summary of accelerated stability test dispensers and ABHS products. (DOCX)
