# Supplementary figures and images for: Presence of unsafe chemical impurities, accelerated evaporation of alcohol, and lack of key labeling requirements are risks and concerns for some alcohol-based hand sanitizers and dispenser practices during the COVID-19 pandemic
Source: PLoS One. 2022 Mar 18;17(3):e0265519. doi: 10.1371/journal.pone.0265519 (PMC8932570; doi:10.1371/journal.pone.0265519)

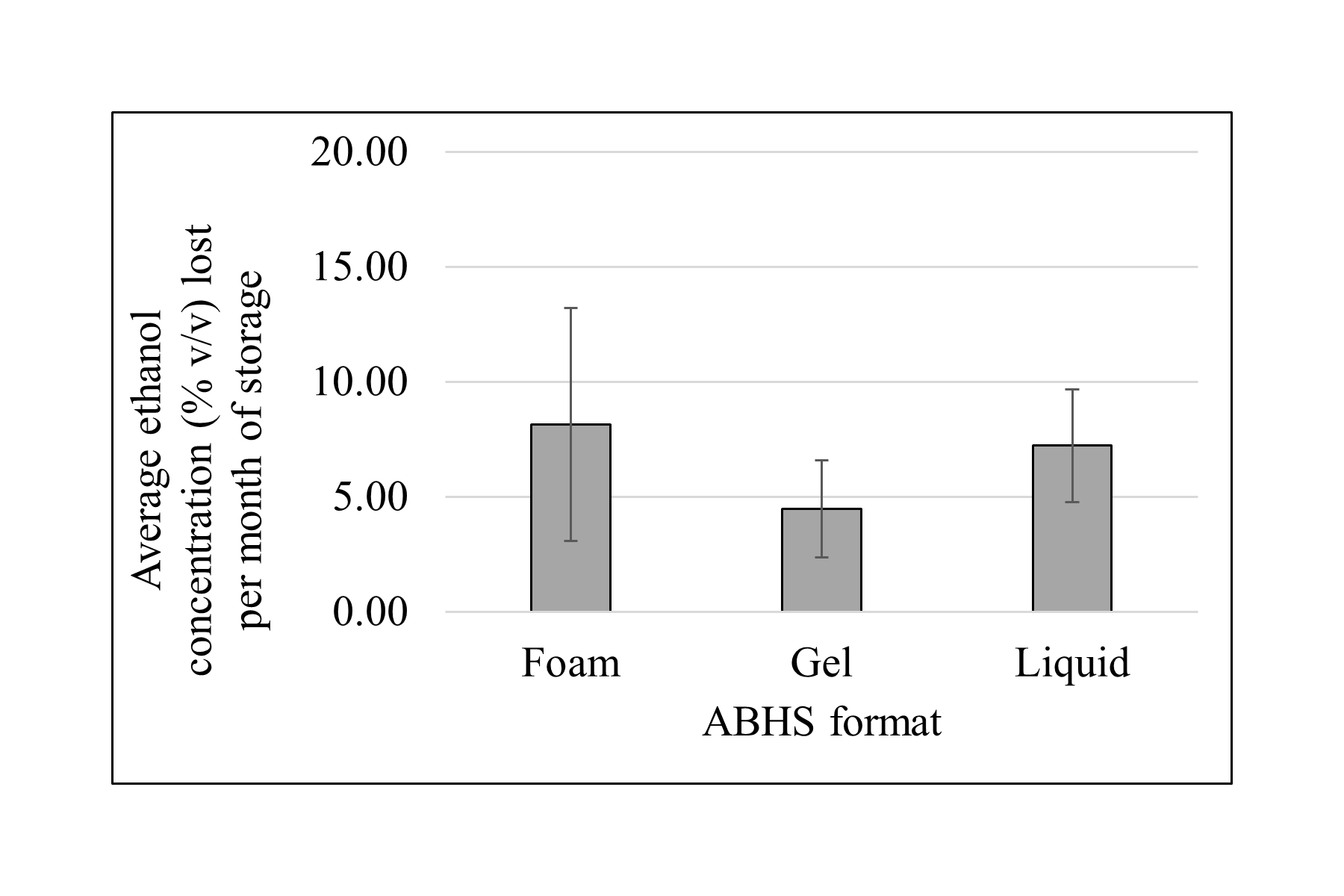

Supplement: S1 Fig — (TIF) [file pone.0265519.s001.tif]
