# Supplementary material for: Presence of unsafe chemical impurities, accelerated evaporation of alcohol, and lack of key labeling requirements are risks and concerns for some alcohol-based hand sanitizers and dispenser practices during the COVID-19 pandemic
Source: PLoS One. 2022 Mar 18;17(3):e0265519. doi: 10.1371/journal.pone.0265519 (PMC8932570; doi:10.1371/journal.pone.0265519)
Supplement: S1 File — (DOCX) [file pone.0265519.s003.docx]

S2 File: Summary of Accelerated Stability Test Dispensers and ABHS Products

**Additional Information on ABHS Dispensers Used for Laboratory-Based Accelerated Stability Studies**

| **Dispenser Designation** | **Dispenser Type** | **Actuation Type** |
| --- | --- | --- |
| A | Stainless Steel Dispenser with an Internal Plastic Refillable Bottle and Plastic Cap | Touch Free |
| B | Plastic Bulk Soap Style Refillable Dispenser with Open Refillable Reservoir | Manual |
| C | Plastic Bulk Soap Style Refillable Dispenser with Open Refillable Reservoir | Touch Free |
| D | Plastic Soap and Sanitizer Dispenser with Internal Plastic Refillable Bottle and Rubber Cap | Touch Free |

**Dispenser Photographs:**


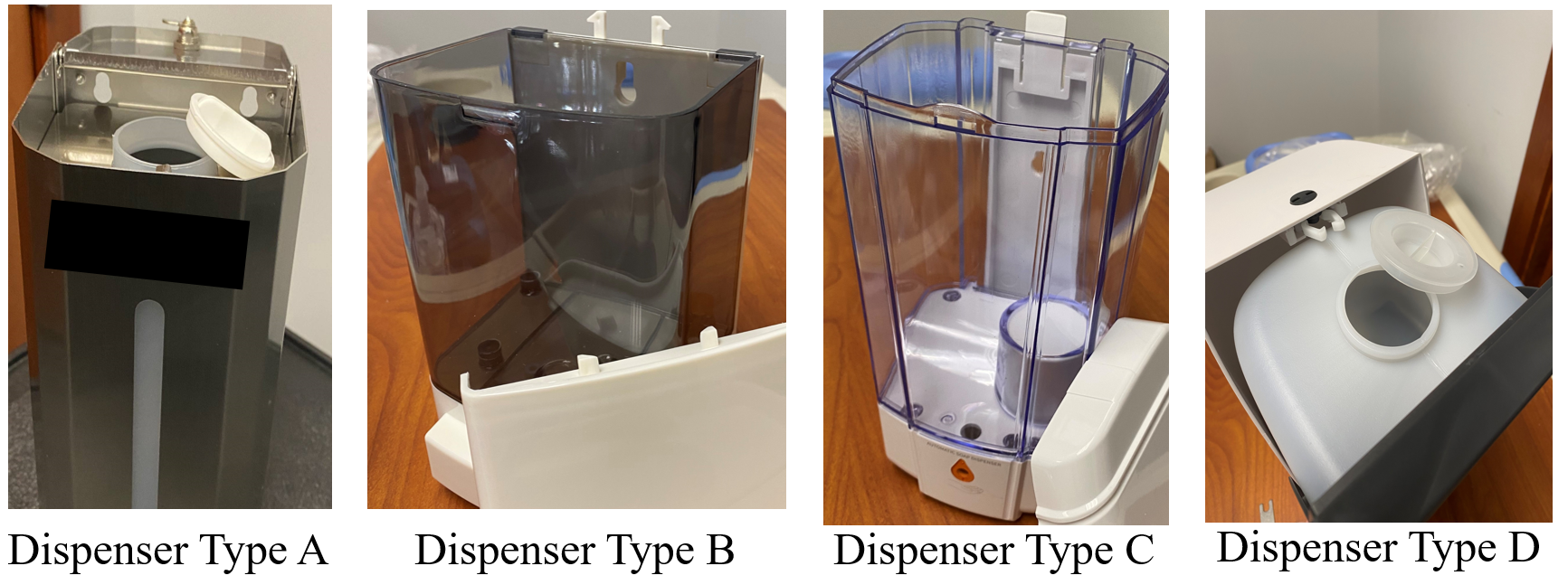


**Additional Information on ABHS Products Used for Laboratory-Based Accelerated Stability Studies**

| **ABHS Designation** | **ABHS Formula Type** | **Ethanol Concentration (Label Indicated)** | **Initial Ethanol Concentration (v/v; Laboratory Tested)** |
| --- | --- | --- | --- |
| A | Foam | 62% | 66.48% |
| B | Foam | 70% | 72.52% |
| C | Foam | 66% | 66.29% |
| D | Gel | 70% | 72.26% |
| E | Gel | 70% | 72.66% |
| F | Gel | 62% | 62.97% |
| G | Liquid (WHO Formula) | 80% | 80.08% |
| H | Liquid (WHO Formula) | 80% | 80.03% |
